# The antioxidant and anti-inflammatory effects of *Carica Papaya Linn.* seeds extract on CCl_4_-induced liver injury in male rats

**DOI:** 10.1186/s12906-021-03479-9

**Published:** 2021-12-30

**Authors:** Nadia Z. Shaban, Sarah M. El-Kot, Olfat M. Awad, Afaf M. Hafez, Ghada M. Fouad

**Affiliations:** 1 Department of Biochemistry, Faculty of Science, Alexandria University, Alexandria, 21511 Egypt; 2 Department of Environmental Studies, Institute of Graduate Studies and Research, Alexandria University, Alexandria, Egypt; 3 Department of Histology and Cell Biology, Faculty of Medicine, Alexandria University, Alexandria, Egypt

**Keywords:** *Carica Papaya Linn.* seeds extract, Liver injury, CCl_4_, Oxidative stress, Inflammation, Apoptosis

## Abstract

**Background:**

Oxidative stress (OS) and inflammation are the central pathogenic events in liver diseases. In this study, the protective and therapeutic role of *Carica Papaya Linn.* seeds extract (SE) was evaluated against the hepatotoxicity induced by carbon tetrachloride (CCl_4_) in rats.

**Methods:**

The air-dried papaya seeds were powdered and extracted with distilled water. The phytochemical ingredients, minerals, and antioxidant potentials were studied. For determination of the biological role of SE against hepatotoxicity induced by CCl_4_, five groups of adult male Sprague-Dawley rats were prepared (8 rats per each): C: control; SE: rats were administered with SE alone; CCl_4_: rats were injected subcutaneously with CCl_4_; SE-CCl_4_ group: rats were administered with SE orally for 2 weeks before and 8 weeks during CCl_4_ injection; SE-CCl_4_-SE group: Rats were administered with SE and CCl_4_ as mentioned in SE-CCl_4_ group with a prolonged administration with SE for 4 weeks after the stopping of CCl_4_ injection. Then, the markers of OS [lipid peroxidation (LP) and antioxidant parameters; glutathione (GSH), superoxide dismutase (SOD), glutathione-S-transferase (GST), glutathione peroxidase (GPx)], inflammation [nuclear factor (NF)-κB, tumor necrosis factor (TNF)-α, interleukin (IL)-6], fibrosis [transforming growth factor (TGF)-β], apoptosis [tumor suppressor gene (p53)], liver and kidney functions beside liver histopathology were determined.

**Results:**

The phytochemical analyses revealed that SE contains different concentrations of phenolics, flavonoids, terpenoids, and minerals so it has potent antioxidant activities. Therefore, the treatment with SE pre, during, and/or after CCl_4_ administration attenuated the OS induced by CCl_4_ where the LP was reduced, but the antioxidants (GSH, SOD, GST, and GPx) were increased. Additionally, these treatments reduced the inflammation, fibrosis, and apoptosis induced by CCl_4_, since the levels of NF-κB, TNF-α, IL-6, TGF-β, and p53 were declined. Accordingly, liver and kidney functions were improved. These results were confirmed by the histopathological results.

**Conclusions:**

SE has protective and treatment roles against hepatotoxicity caused by CCl_4_ administration through the reduction of OS, inflammation, fibrosis, and apoptosis induced by CCl_4_ and its metabolites in the liver tissues. Administration of SE for healthy rats for 12 weeks had no adverse effects. Thus, SE can be utilized in pharmacological tools as anti-hepatotoxicity.

## Background

Liver diseases are an extremely predominant disease and one of the most important causes of death worldwide. Liver diseases are triggered by consecutive exposure to xenobiotics such as carbon tetrachloride (CCl_4_) and drugs as paracetamol [1, 2]. Xenobiotics can cause severe hepatocyte injuries associating with acute or chronic hepatic inflammation. Several studies showed that oxidative stress (OS) and inflammation are the most important pathogenic events in liver diseases regardless of etiology [3].

CCl_4_ is a colorless liquid with a sugary odor. It is used as a solvent, reagent in the chemical synthesis, fabric-spotting fluid, dry-cleaning agent, fire extinguisher fluid, and grain fumigant [4]. It is emitted to the environment predominantly through direct releases to air with lower amounts discharged to water and soil [4]. CCl_4_ is a cytotoxic agent where it is rapidly absorbed by the liver and then metabolized inside the cells by cytochrome (CYP) 450 into active metabolites (trichloromethyl radical; CCl_3_• and trichloromethyl peroxyl radical; CCl_3_OO•) [5, 6]. These free radicals induce OS “an imbalance between the production and the elimination of reactive species [reactive oxygen species; ROS and reactive oxygen species; RNS]”. OS induces lipid peroxidation (LP), protein oxidation, and DNA damage leading to various diseases including Liver diseases [7].

Different chemical drugs are utilized for the treatment of liver diseases but they can cause severe side effects in the human body [8]. For instance, paracetamol, a well-known antipyretic drug, can cause hepatotoxicity [9]. Therefore some natural product-derived drugs, a hallmark of modern pharmaceutics, are used instead of several types of synthetic drugs. So, they include vitamin A, quinine, digoxin, theophylline, penicillin G, morphine, paclitaxel, vincristine, cyclosporine, and doxorubicin which used in the treatment of many diseases such as breast cancer, liver diseases, leishmaniasis, lymphatic filariasis, etc. where, natural products have a wide range of diversity of multi-dimensional chemical structures [10, 11].

*Carica papaya Linn.* (*C. Papaya*) is commonly called as paw-paw and it belongs to the class Magnoliopsida, the order Brassicales, the family Caricaceae, Genus *Carica L.* It is consumed, in two forms natural or processed, to share the nourishing value [12, 13]. *C. Papaya* seeds constitute represent about 15 to 20% of the fruit net weight [14]. The seeds oil is rich in oleic, palmitic, linoleic, and stearic acid. Also, it contains myristic acid, palmitoleic acid, linolenic acid, arachidic acid, and gadoleic acid in small amounts [14]. The biological activities of the seeds oil attribute to the existence of alkaloids, flavonoids, saponins, sarcotesta, fiber, and other active phytocomponents [15]. Some studies showed that seeds extract is characterized by its antibacterial, anti-fertility, antiparasitic and anti-implantation activities as well as its efficiency as abortifacient [16]. Therefore, the present study was carried out to investigate the protective and therapeutic role of *C. Papaya* seeds extract (SE) against hepatotoxicity induced by CCl_4_. The study focused on the determination of the markers of OS, inflammation, fibrosis, apoptosis, lipid profile, liver function, and its histological examination beside kidney functions. Additionally, the phytochemical components and characterization of seeds extract were studied.

## Methods

### Chemicals and reagents

Folin-Ciocalteau reagent, gallic acid (GA), ursolic acid (UA), rutin (RU), catechin, 2,4 dinitrophenyl hydrazine (DNPH), 2,2 diphenyl-1-picrylhydrazyl (DPPH), butylatedhydroxytoluene (BHT), 5, 5′, Dithiobis-2-nitrobenzoic acid (DTNB), 2,2-azinobis (3-ethylbenzothiazoline-6-sulfonic acid) (ABTS), CCl_4_ (reagent grade, 99.9%) and GSH were obtained from Sigma-Aldrich, St Louis, MO, USA. thiobarbituric acid (TBA) was obtained from El-Nasr Pharmaceutical Chemicals Co. (Alex., Egypt). Ascorbic acid (Asc) was obtained from Riedel-de Haën, Germany. Biozol reagent was obtained from Invitrogen, CA, USA. SYBER Green 1-step qRT-PCR Kit was purchased from Thermo Scientific, USA. Primers for nuclear factor kappa B (NF-κB), tumor necrosis factor (TNF)-α, interleukin (IL)-6, transforming growth factor (TGF)-β and the tumor suppressor gene p53 were purchased from Bioneer, Korea. Kits for alanine aminotransferase (ALT), aspartate aminotransferase (AST), alkaline phosphatase (ALP), total protein (TP), albumin, creatinine, urea, high-density lipoprotein cholesterol (HDL-c) low-density lipoprotein cholesterol (LDL-c) and triglycerides (TG) were purchased from Biodiagnostic, Cairo, Egypt.

### Plant

*C. Papaya* fruit, belongs to the Caricaceae family, was obtained from Nubaria, Behera, Egypt. The fruits were carefully chosen for its uniformity, shape, size, color, and absence of fungal infection.

### Preparation of SE aqueous extract

*C. Papaya* seeds were separated from the flesh manually, washed with tap water, and dried in shadow at 22 ± 3 °C for 4 weeks. The dried seeds were crushed pulverized into fine powder by a grinder (Moulinex, France). Then, the powder was soaked in distilled water (1:10 w/v) for 72 h at 4 °C and filtered using Whatman filter paper where the extraction process was repeated 3 times. The total filtrates were lyophilized (Virtis 248625 Freeze Dryer; USA) producing a fine sweet-smelling and chocolate color solid residue. The solid residue was stored in an air tight dark bottle at 4 °C till use [17].

### Characterization of SE

#### Total phenolic content (TPC)

The TPC was assayed colorimetrically as GA equivalent (eq) in mg/g SE, using GA as standard and Folin-Ciocalteau reagent. The absorbance of the obtained color solution was recorded at 750 nm [18].

#### Total flavonoids (TFC)

The TFC was quantified as mg RU eq/g SE, using 10% aluminum chloride and 5% sodium nitrite solutions, since the absorbance of the colored product was determined at 510 nm [19].

#### Tannin content

Tannins were determined colorimetrically as mg catechin eq/g SE using 2% vanillin dissolved in methanol, where the colored solution formed was read at 500 nm [20].

#### Analysis of phenolic and flavonoid compounds using high performance liquid chromatography (HPLC)

The HPLC analysis was performed according to [21, 22]. Two hundred microliters of SE was separated on Eclipse XDB–C18 column (150 mm × 4.6 mm, 5 μm; Agilent Technologies, Palo Alto, CA, USA). The separation flow rate was 0.75 mL/min at 320 nm, using mobile phase 2-propanol: acetonitrile: 1% formic acid (8:22:70) at pH 2.5.

#### Triterpenoids and Asc content

Triterpenoids were determined as mg UA eq/g SE using 5% vanillin dissolved in glacial acetic acid and the colored product was measured at 520 nm [23]. The Asc concentration was estimated in SE using the standard curve by using DNPH; the absorbance of the colored solution was read at 520 nm [24, 25].

### Antioxidant potentials of SE

The antioxidant activities of SE were evaluated using different antioxidant methods. The total antioxidant capacity (TAC), antiradical potentials (DPPH and anti-ABTS+), and ferric reducing antioxidant power (FRAP) were assayed. TAC of SE was measured by adding 1.9 mL of the reagent solution (0.6 M H_2_SO_4_, 28 mM sodium phosphate and 4 mM ammonium molybdate) to 100 μL of SE or serial dilution of Asc (0–1 mg/mL), the absorbances were measured at 695 nm after incubation for 90 min at 95 °C [26]. The effect of SE on DPPH radical was performed according to the modified standard method of Blois [27], in which 200 μL of DPPH was mixed with 1 mL of different concentrations (0-1 mg/ mL) of SE, Asc or ethanol as a control, then the absorbances of the colored products were read at 490 nm after incubation in the dark for 20 min at 25 °C. The IC50 (50% inhibitory concentration) values of both SE and Asc were evaluated using the following equation: inhibition (%) = (1 – (A_extract_ / A_control_)) × 100 [27]. The ABTS radical cation was monitored through the addition of different concentrations of SE or Trolox to 1 mL of ABTS working reagent (7 mM ABTS water solution and 140 mM potassium persulphate), the absorbances were read at 734 nm after incubation for 2.5 min at 25 °C to be used for the calculation of ABTs % inhibition as mentioned above [28]. The FRAP of the SE was determined by assessing the ability of the plant to reduce FeCl_3_ solution and the absorbance was monitored at 700 nm as defined by Oyaizu [29], A graph of absorbances vs. different concentrations of the SE or Asc were plotted to calculate the FRAP IC50 value.

### The biological effect of SE on hepatotoxicity induced by CCl_4_

#### Animals

**Fig. 1:**
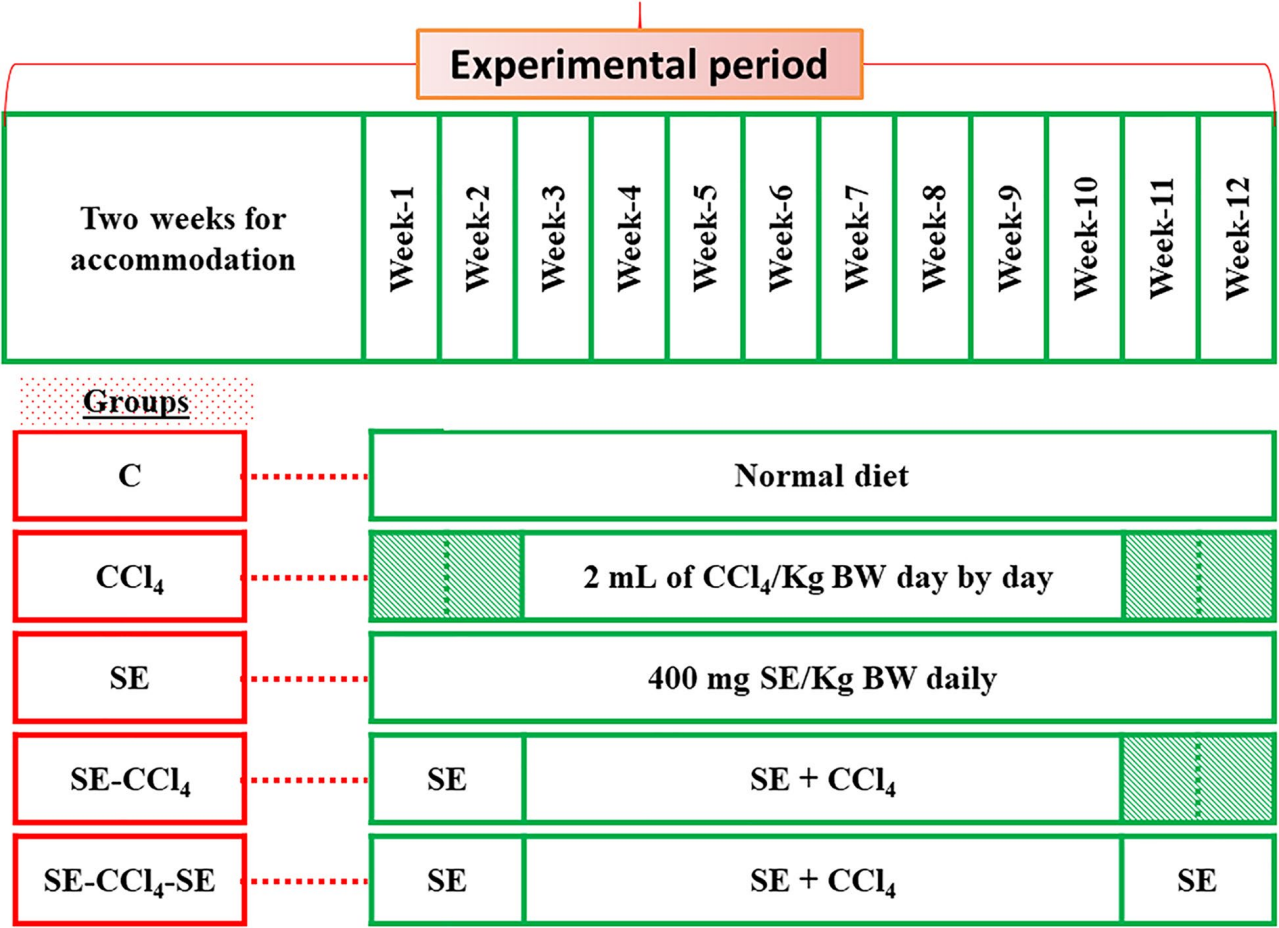
The experimental design

The experimental animals were accommodated and handled in accordance with the Ethical approval of the ALEXU-IACUC (Institutional Animal Care and Use Committee) (code NO. AU 04 20 12 26 2 01, Alexandria University, Faculty of Science) and the work has been reported in accordance with the ARRIVE guidelines (Animals in Research: Reporting In Vivo Experiments) [30]. Forty adult male Sprague-Dawley rats (60 - 70 days old; 150 – 170 g body weight) were obtained from Faculty of Agriculture, Alexandria University, Egypt. The rats were examined for health status, housed and handled under ethical conditions, according to the international rules of animal care. The ambient temperature was 25 ± 0.5 °C, with 12 hs light/dark cycle [31, 32]. All rats were left for 2 weeks for acclimatization and then divided into five groups, eight rats each (Fig. 1). **Control group (C):** Healthy rats without any treatment; **CCl**_4 _**group:** Rats were injected subcutaneously with 2 mL of CCl_4_ dissolved in olive oil/kg body weight (BW) day by day for 8 weeks [33]; **SE group:** Rats were administered with SE orally (using oral gavage) with 400 mg of SE dissolved in 1 mL distal water/kg BW/day for 12 weeks [34]; **SE-CCl**_4 _**group:** Rats were orally administered with SE (as in SE group) and at the beginning of the 3rd week, they were injected with CCl_4_; **SE-CCl**_4_**-SE group:** Rats were given SE and CCl_4_ as in SE-CCl_4_ group with continuous administration of SE for 2 weeks after the stopping of CCl_4_ injection.

At the end of the experimental period, feeding was stopped 12 hs before dissection. Rats were anaesthetized using carbon dioxide gas and then scarified. Blood samples were collected from rat portal veins in empty tubes, kept at room temperature for 15 min, centrifuged at 1000 xg for 10 min. The sera were stored at − 20 °C until used for the determination of ALT, AST, ALP, TP, albumin, urea, creatinine, LDL-c, HDL-c, and TG. Livers were separated immediately, washed with cold saline solution (0.9% NaCl), divided into three parts. The first part was fixed in 10% formalin and used for the histological studies. The second part was kept at − 80 °C in RNA later solution till used for the detection of inflammation, fibrosis and apoptosis. The third part was homogenized in cold 0.1 M sodium phosphate buffer (pH 7.4) containing saline solution. The liver homogenate was centrifuged at 10000 xg for 20 min and the supernatant was kept at − 80 °C till used for the determination OS markers.

### Histopathological examinations of liver tissues

The liver tissues were fixed, processed, and embedded in paraffin wax according to Griffith and Farris. Portions of 5 μm in thickness were cut and stained with hematoxylin and eosin (H & E) stain [35].

### Biochemical assays

#### Assessments of OS markers

In liver homogenates, the oxidant MDA (the end product of LP) was assayed [31, 36, 37]. Also, nitric acid (NO) as oxidant was measured colorimetrically by recording the level of the nitrite using the Griess reagent [38].

All antioxidants were determined in liver homogenates. The GSH was determined by the reaction of DTNB with GSH giving a yellow product which measured at 412 nm [39]. GSH is expressed as mg/mg protein. GSR activity relies on the oxidation of NADPH in the homogenates in the presence of GSSG [40]. GSR is expressed as μmol/min/mg protein. The GST assay was determined via the reaction of GST substrate (p-nitrobenzyl chloride) with GSH to give a conjugate product which read at 310 nm [41]. The SOD was evaluated via an indirect method [42]. The unit of activity is defined as the amount of enzyme that inhibits the rate of pyrogallol autoxidation under standard conditions, and the change in absorbance was measured in 2 min at 420 nm. SOD is expressed as U/mg protein. t-GPx was determined by measuring the NADPH oxidation in the homogenates in the presence of GSH and cumene hydroperoxide at 340 nm [43].

### Assessments of inflammatory, apoptotic and fibrotic markers

The quantitative gene expression of the markers of inflammation (NFĸB, TNF-α, IL-6), apoptosis (p53), and fibrosis (TGF-β) were determined in the RNA reaction mixtures of the liver tissues using quantitative reverse transcriptase PCR (qRT-PCR).

#### RNA extraction

The frozen liver tissues were cut into small slices and transferred to an Eppendorf tube containing 1 mL Biozol reagent [44, 45]. The extraction protocol was applied as indicated by the manufacturer guidelines. In brief, the liver tissues were homogenized using forceps, and incubated at 4 °C for 15 min. One milliliter glycogen was added to the homogenate, mixed well, chloroform was added, and incubated at 4 °C for 15 min. The mixture was centrifuged, and the aqueous layer was transferred to nuclease-free Eppendorf tube. An equal volume of cold isopropyl alcohol was added to the aqueous layer to precipitate the RNA content. The precipitated RNAs were washed, treated with DNAase to get rid of DNA, and then stored at -80 °C till used for the determination of the gene expressions of NFкB, TNF-α, IL-6, TGF-β, and p53 using a quantitative real-time polymerase chain reaction (qRT-PCR).

#### qRT-PCR

Using SYBER Green 1-step qRT-PCR Kit, gene expression of the target genes and the reference gene were quantified by qRT-PCR System using the specific primers sequences (Forward/Reverse) as shown in Table 1 [37, 46–48]. The qRT-PCR was performed in a reaction mixture of 10 μL (as total volume) using 5 μL 1-step QPCR SYBER mix (1X), 0.1 μL verso enzyme mix, 0.5 μL RT-enhancer, 0-2.9 μL water (PCR grade), 0.5 μL forward and reverse primers (10 pm), and 0.5-3.4 μL RNA template. qRT-PCR program was carried out as one cycle of cDNA synthesis at 50 °C for 15 min, one cycle of Thermo-start enzyme activation at 95 °C for 15 min and followed by denaturation at 95 °C in 40 cycles for 15 s, annealing at 60 °C for 1 min and extension at 72 °C for 30 s.

**Table 1:** The forward and reverse sequences of the gene’s primers used in qRT-PCR technique

Gene name	Sequences (Forward/Reverse)
β-actin	F: GTGGGCCGCTCTAGGCACCAA
	R: CTCTTTGATGTCACGCACGATTTC
NF-ĸB	F: CTGGCAGCTCTTCTCAAAGC
	R: CCAGGTCATAGAGAGGCTCAA
TNF-α	F: ATGAGCACAGAAAGCATGATCCGCG.
	R: CCCTTCACAGAGCAATGACTCCAAA
IL-6	F: GATGCTACCAAACTGGATATAATC
	R: GGTCCTTAGCCACTCCTTCTGTG
p53	F: CACAGTCGGATATGAGCATC.
	R: GTCGTCCAGATACTCAGCAT
TGF-β	F: CGGGAAGCAGTGCCAGAA.
	R: TCCACAGTTGACTTGAATCTC

*NF-κB:* Nuclear factor kappa B, *TNF-α:* Tumor necrosis factor-α, *IL-6:* Interleukin-6, *p53:* Tumor suppressor gene, *TGF-β:* Transforming growth factor-β

### Liver functions and lipid profile

Liver function tests (ALT, AST, and ALP activities beside TP and albumin levels) and the lipid profile tests (TG, LDL-c, and HDL-c) were assayed in serum using commercial kits [49–53].

### Kidney function tests

Kidney functions including urea and creatinine levels were determined using kits [54, 55].

#### Statistical analysis

All data were presented as mean (X) ± standard deviation (SD). The statistical analysis was performed by using one-way analysis of variance (ANOVA). Comparisons between the means of various treatment groups were analyzed using the post hoc analysis (Duncan’s test) by SPSS (Statistical Package for Social Sciences) software, version 25. The statistical difference values were considered at *p* < 0.05.

## Results

### Characterization of SE

#### Phytochemical and mineral contents of SE

**Fig. 2:**
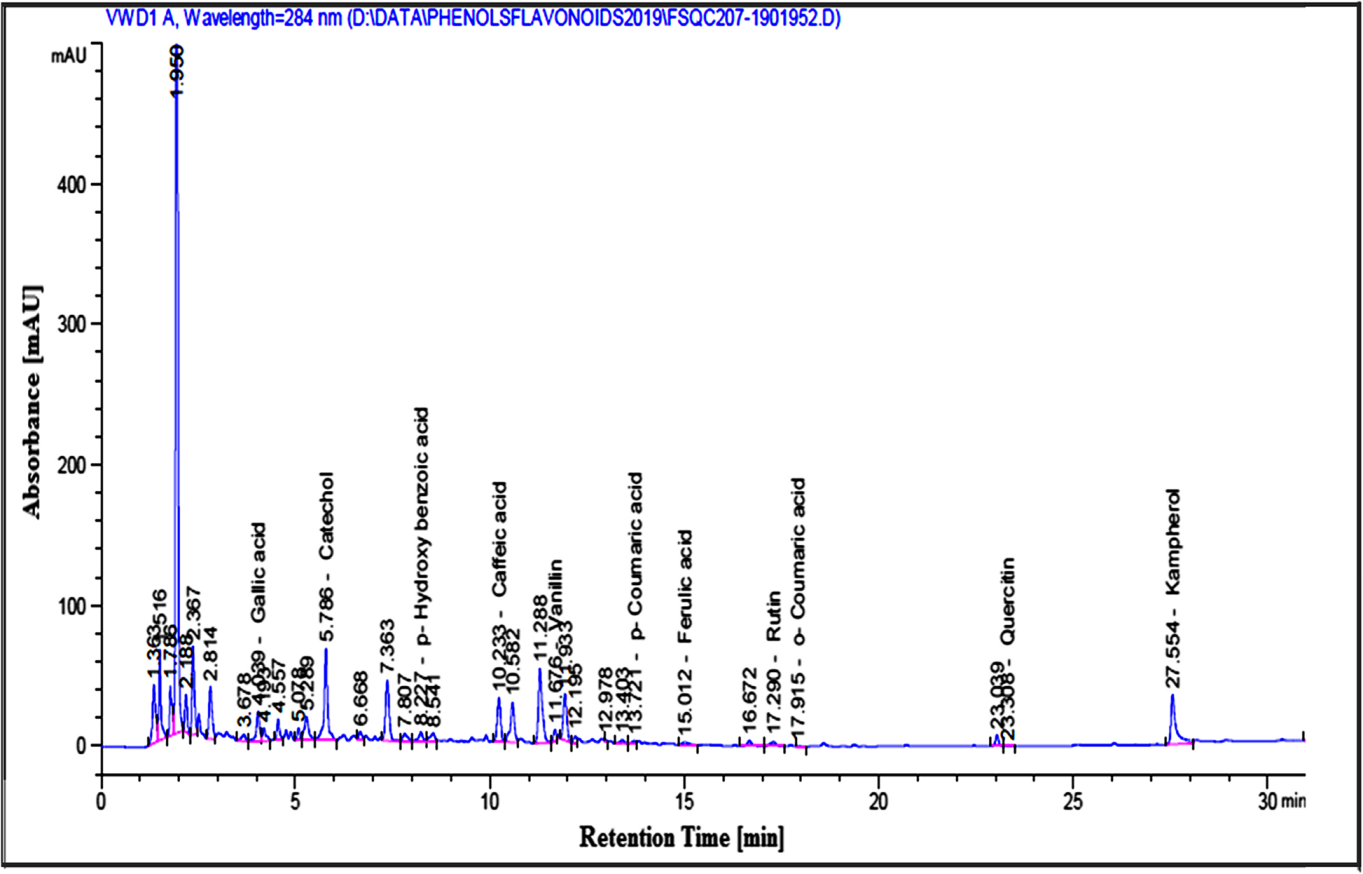
HPLC analysis of phenolic and flavonoid compounds of SE

The results in Table 2 represent the phytochemical composition of SE where these results showed that SE has considerable amounts of phenolics, flavonoids, tannins, triterpenoids, and Asc. Also, the HPLC analysis recognized different phenolic and flavonoid compounds in SE by comparing the retention time with the known phenolic and flavonoid standards (Fig. 2). Table 2 shows that SE contains different minerals which were arranged according to their concentration orders; Ca > K > S > Mg > Na > Fe > Zn > Mn > Cu > Mo > Se > Ni > Co.

**Table 2:** Phytochemicals and minerals composition of SE

**Phytochemical’s ingredients**			
**compound**	**Concentration**	**compound**	**Concentration**
**TPC (mg GA eq/g SE)**	31.32 ± 0.00	**Triterpenoids (mg UA eq/g SE)**	73.84 ± 0.00
**TFC (mg RU eq/g SE)**	6.32 ± 0.00	**Ascorbic acid (mg/g SE)**	0.112 ± 0.02
**Tannins (mg catechin eq/g SE)**	0.53 ± 0.01	**TAC (μg BHT eq/g SE)**	1.59 ± 0.02
**Element’s composition**			
**Elements name**	**Concentration (mg/100 g tissue)**	**Elements name**	**Concentration (mg/100 g tissue)**
**K**	1931 ± 0.01	**Mn**	3.090 ± 0.01
**Ca**	1161 ± 0.00	**Cu**	1.440 ± 0.00
**S**	534.0 ± 0.01	**Mo**	0.225 ± 0.00
**Mg**	426.8 ± 0.01	**Se**	0.100 ± 0.00
**Na**	160.6 ± 0.00	**Ni**	0.025 ± 0.00
**Fe**	9.160 ± 0.00	**Co**	0.005 ± 0.00
**Zn**	7.100 ± 0.01		

Results are presented as Mean ± SD (*n* = 3)

*TPC:* Total phenolic compounds, *GA:* Gallic acid, *TFC:* Total flavonoid compounds, *RU:* Rutin, *UA:* Ursolic acid, *TAC:* Total antioxidant capacity, *BHT:* Butylated hydroxytoluene

#### Antioxidant potentials

**Fig. 3:**
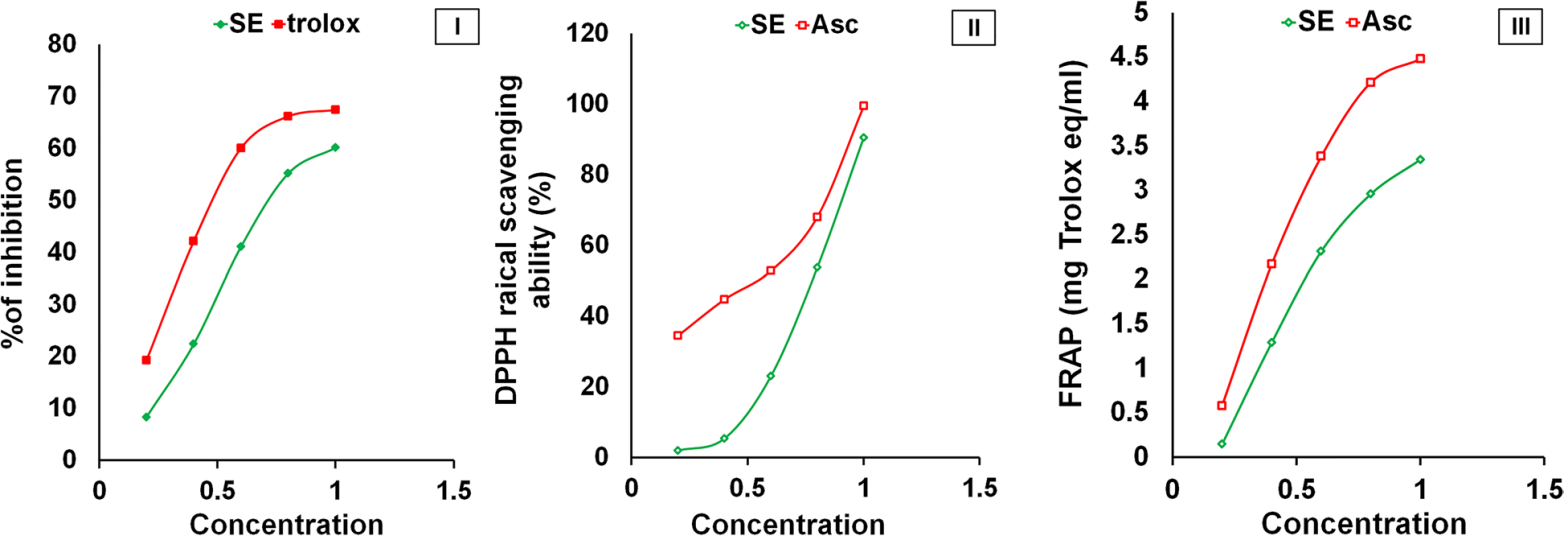
The antioxidant activities of SE compared to Asc or Trolox. (I) DPPH scavenging activity, (II) ABTS scavenging activity, and (III) FRAP. The results are presented as mean ± SD (*n* = 3)

The results showed that the TAC of SE were 158.9 mg BHT eq/g (Table 2). The ABTs+ and DPPH scavenging activities were determined using IC50 and expressed in terms of mg/ml. The values of IC50 indirectly proportionate to the scavenging activity of SE. The IC50 values of DPPH radical scavenging activity, anti-ABTs+ activity and FRAP of SE were 0.907, 0.695, 9.899 mg eq/mL (Fig. 3). The scavenging activities of the SE and Asc against DPPH and ABTs+ showed that SE inhibited the ROS in a concentration-dependent manner.

### Protective and therapeutic role of SE against rat hepatotoxicity

#### Liver histopathology of different studied groups

**Fig. 4:**
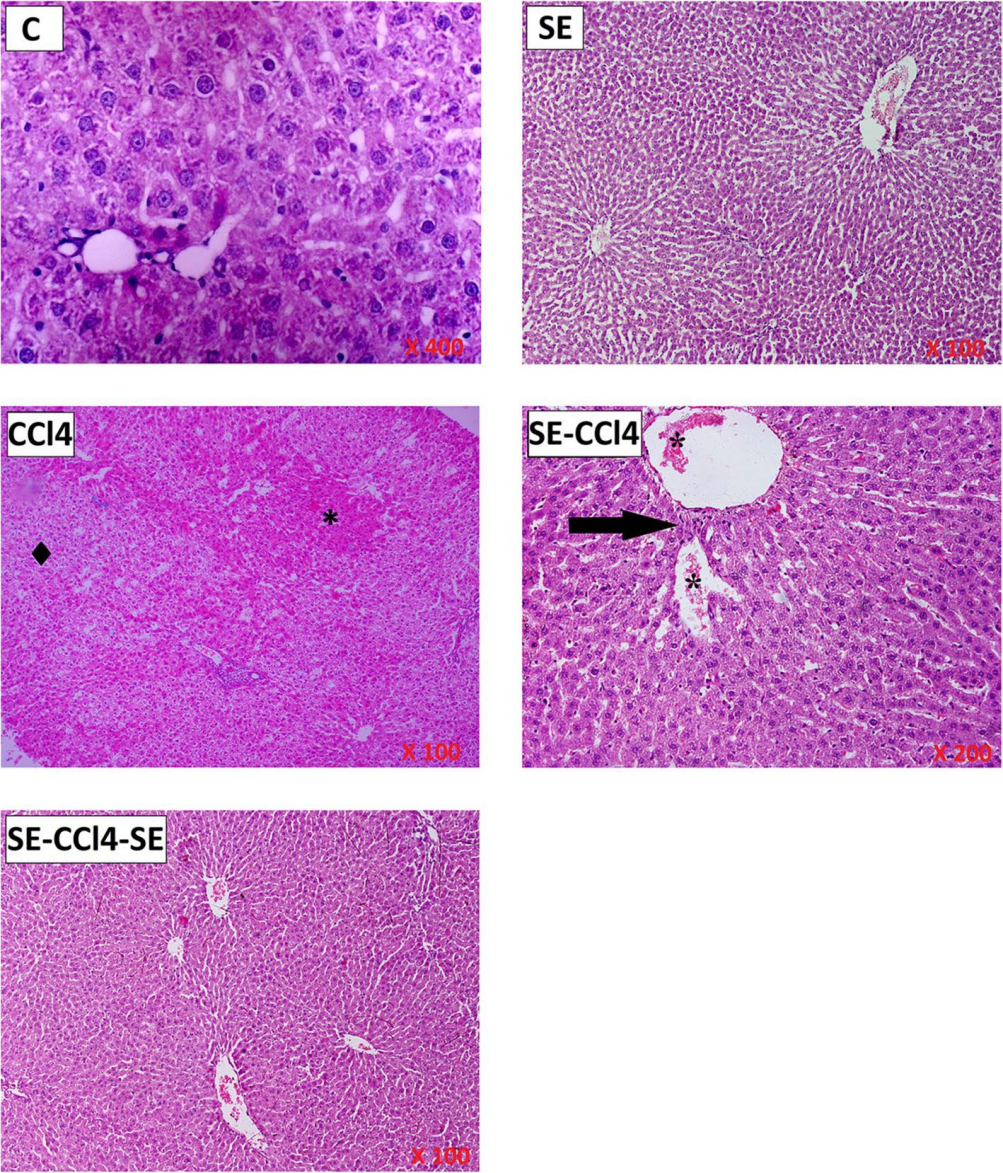
Representative microscopic images of H & E stained liver sections of all studied groups. C group: liver demonstrating the normal organization of hepatocytes and hepatic lobules. SE group: Hepatocytes showing of rat liver with cytoplasmic vacuolation in most cells. CCl_4_ group: revealing focal areas with eosinophilic hepatocytes (*) alternating with areas of pale vacuolated hepatocytes (•). Some portal tracts PT show dense cellular infiltration and fibers in between. There are groups of degenerated hepatocytes with loss of nuclei. SE group: liver section showing preserved normal liver architecture (arrow). SE-CCl_4_: regaining the eosinophilic granular appearance of the cytoplasm in hepatocytes. The central vein and portal vein tributary in the portal tract area abnormally approximated denoting persistently disrupted the architecture of the liver lobule (arrow). They show dilatation and engorgement with blood cells in the lumen (*). SE-CCl_4_-SE group: showing apparent recovery of the architecture of hepatic lobule. Normal hepatocytes arranged in cords radiating from the central vein with clear sinusoids of average size

The histopathological results of the SE group showed that there is no changes in the liver histology when compared with the control group indicating that SE did not induce any apparent alterations in the hepatic parenchyma liver cells (Fig. 4). CCl_4_-group showed disperse focal degenerative changes in the liver parenchyma appearing as focal pale areas with hepatocyte vacuolation, steatosis, cell degeneration, and empty cells with dark pyknotic nuclei alternating with foci. On the level of individual cells, groups of pale degenerated hepatocytes were seen with individual intact eosinophilic hepatocytes in-between (Fig. 4). Treatment with SE before, during and/or after CCl_4_ administration (SE-CCl_4_ & SE-CCl_4_-SE groups) resulted in relative improvements of the histology of hepatocytes lesion induced by CCl_4_ (Fig. 4).

#### SE reduced hepatotoxicity induced by CCl_4_

The data of liver functions are recorded in Table 3. The administration of SE for healthy rats had no remarkable changes on the liver functions compared to the control group. CCl_4_ administration (CCl_4_-group) caused a significant elevation (*p* < 0.05) in the activities of ALT, AST, and ALP with a significant decline (*p* < 0.05) in TP and albumin levels as compared to the control rats. The administration of SE pre, during and/or after CCl_4_ injection decreased ALT, AST, and ALP activities significantly (*p* < 0.05) and elevated TP and albumin levels significantly (*p* < 0.05) as compared to the CCl_4_ group.

**Table 3:** Effect of SE treatment on liver function tests, kidney function tests and lipid profile parameters

Group / Parameters	C	SE	CCl_**4**_	SE-CCl_**4**_	SE-CCl_**4**_–SE
**ALT (U/L)**	16.0 ± 2.1^a^	15.7 ± 1.6^a^	91.0 ± 3.5^b^	31.2 ± 2.1^c^	28.3 ± 1.8^d^
**AST (U/L)**	63.2 ± 2.4^a^	65.3 ± 4.2^a^	226.9 ± 4.6^b^	73.4 ± 6.1^c^	74.7 ± 7.7^c^
**ALP (U/L)**	67.2 ± 2.1^a^	71.6 ± 4.8^a^	171.0 ± 4.2^b^	113.6 ± 5.2^c^	103.8 ± 7.2^d^
**TP (g/dl)**	54.4 ± 4.4^a^	53.6 ± 3.6^a^	5.08 ± 0.8^b^	31.4 ± 1.6^c^	42.9 ± 1.4^d^
**Albumin (g/dl)**	36.1 ± 0.68^a^	35.8 ± 0.71^a^	5.8 ± 1.60^b^	14.2 ± 0.66^c^	21.4 ± 2.49^d^
**Creatinine (mg/dl)**	0.38 ± 0.05^a^	0.37 ± 0.02^a^	1.83 ± 0.08^b^	1.04 ± 0.04^c^	0.73 ± 0.04^d^
**Urea (mg/dl)**	22.5 ± 1.7^a^	23.3 ± 1.4^a^	98.9 ± 3.5^b^	71.6 ± 6.6^c^	40.2 ± 3.3^d^
**LDL (mg/dl)**	52 ± 0.7^a^	31 ± 0.3^b^	832 ± 1.6^c^	355 ± 1.9^d^	249 ± 1.1^e^
**HDL (mg/dl)**	40.6 ± 1.8^a^	39.4 ± 1.8^a^	13.8 ± 2.3^b^	27.5 ± 2.7^c^	33.8 ± 2.3^d^
**TG (mg/dl)**	113 ± 6.1^a^	115 ± 9.4^a^	412 ± 5.8^b^	262 ± 9.7^c^	216 ± 8.9^d^

The results are shown as mean ± SD (*n* = 8). Different letters for the same parameter are significantly different at *p < 0.05*

*C group* Oral administration with water, *CCl*_*4 *_*group* Rats injected with CCl_4_, *SE group* Rats received SE only, *SE-CCl*_*4 *_*group* Rats received SE before and during CCl_4_ administration, *SE-CCl*_*4*_*-SE group* Rats received SE before, during and after CCl_4_ administration

#### Levels of lipid profile

Table 3 shows that SE administration to healthy rats had a significant (*p* < 0.05) decline in LDL-c level with non-significant (*p* > 0.05) changes in TG and HDL-c compared with the control group. TG and LDL-c levels were significantly (*p* < 0.05) increased but HDL-c was declined significantly (*p* < 0.05) in rats injected with CCl_4_ as compared with the C group. In contrast, the administration of SE pre, during and/or after CCl_4_ injection caused significant (*p* < 0.05) reductions in TG and LDL-c levels with a significant (*p* < 0.05) elevation in HDL-c level compared to CCl_4_ group.

#### Treatment with SE reduced OS induced by CCl_4_

**Fig. 5:**
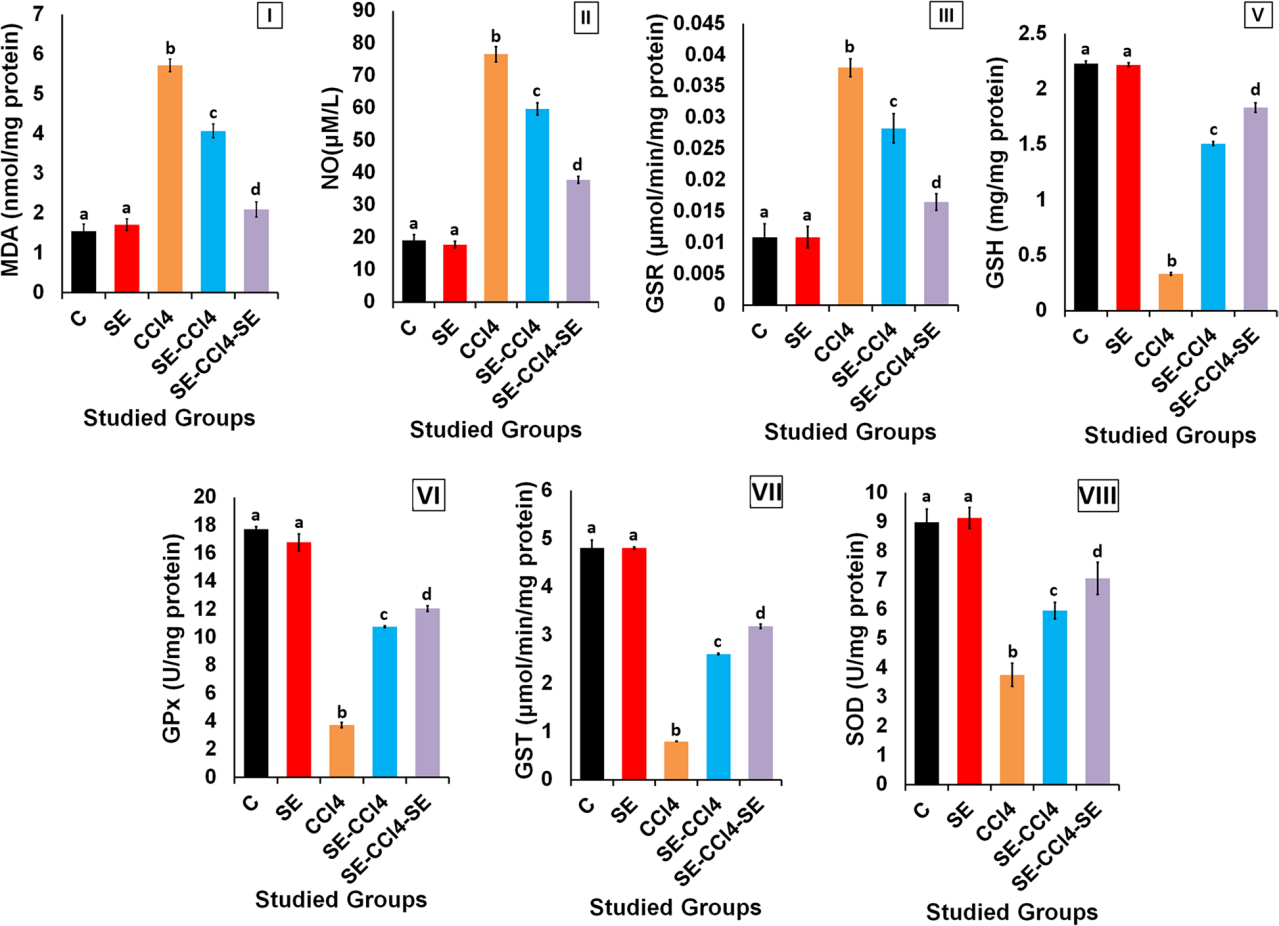
Effect of SE treatment on OS induced by CCl_4_ in liver. (I) MDA levels, (II) NO levels, (III) GSR activities, (IV) GSH levels, the activities of; (V) GPx, (VI) GST, (VII) SOD. C group: oral administration with water; CCl_4_ group: rats injected with CCl_4_. SE group: rats received SE only. SE-CCl_4_ group: rats received SE before and during CCl_4_ administration. SE-CCl_4_-SE group: rats received SE before, during and after CCl_4_ administration. The results are shown as mean ± SD (*n* = 8). Different letters for the same parameter are significantly different at *p < 0.05*

The healthy rats administered with SE only showed non-significant (*p* > 0.05) changes in the levels of MDA, NO and GSH and the activities of GSR, GST, SOD, and GPx compared to the control group (Fig. 5). However, CCl_4_ injection significantly (*p* < 0.05) decreased GSH level and the activities of GST, SOD, and GPx but increased the MDA level and GSR activity as compared with the control group. The SE administration pre, during and/or after CCl_4_ injection markedly improved GSH level and the activities of GST, SOD, and t-GPx, while MDA level and GSR activity were significantly (*p* < 0.05) decreased as compared with CCl_4_-group.

#### SE treatment reduced inflammation induced by CCl_4_

**Fig. 6:**
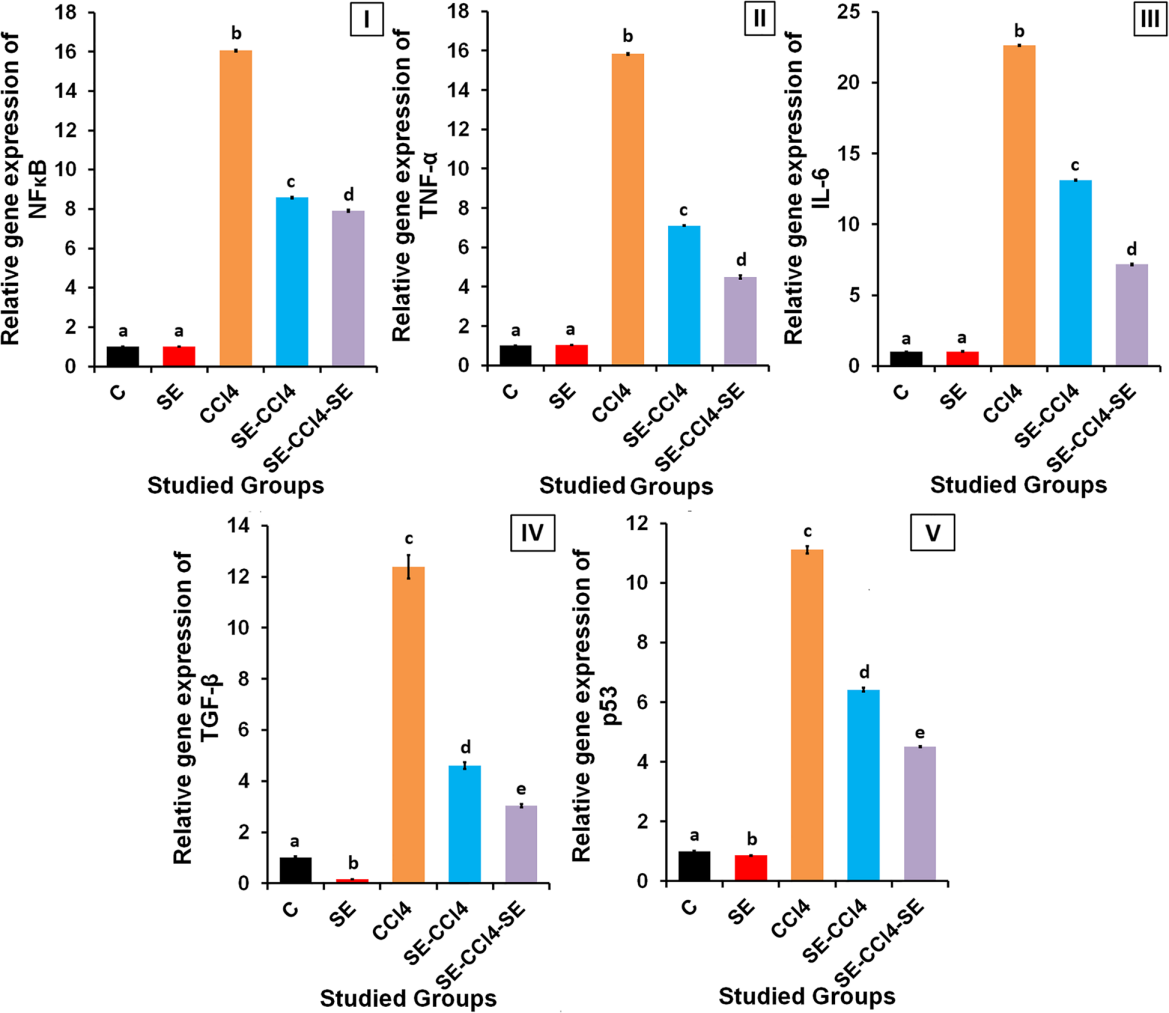
Effect of SE treatment on inflammation, fibrosis and apoptosis induced by CCl_4_ in liver. The relative gene expression of; (I) NF-KB, (II) TNF-α, (III) IL-6, (IV) TGF-β, (V) p53. C group: oral administration with water; CCl_4_ group: rats injected with CCl_4_. SE group: rats received SE only. SE-CCl_4_ group: rats received SE before and during CCl_4_ administration. SE-CCl_4_-SE group: rats received SE before, during and after CCl_4_ administration. The results are shown as mean ± SD (*n* = 8). Different letters for the same parameter are significantly different at *p < 0.05*

The administration of healthy rats with SE showed non-significant (*p* > 0.05) changes in the relative gene expression of NF-κB, TNF-α, and IL-6 at mRNA levels compared to the control group (Fig. 6). The administration of CCl_4_ significantly (*p* < 0.05) increased the relative gene expressions of NF-κB, TNF-α, and IL-6 compared to the control group. However, the treatment with SE pre, during and/or after CCl_4_ injection caused significant declines (*p* < 0.05) in the NF-κB, TNF-α and IL-6 gene expressions compared to the CCl_4_-group.

#### SE treatment reduced the fibrosis and apoptosis induced by CCl_4_

SE administration to healthy rats caused significant (*p* < 0.05) declines in the relative gene expressions of TGF-β and p53 when compared with the control group (Fig. 6). CCl_4_ administration up-regulated the gene expression of TGF-β and p53 significantly (*p* < 0.05) as compared with the control rats. While, SE treatment pre, during and/or after CCl_4_ injection down-regulated the gene expressions of TGF-β and p53 significantly (*p* < 0.05) as compared with the CCl_4_-group.

#### SE reduced nephrotoxicity induced by CCl_4_

Table 3 shows that administration of SE for healthy rats exhibited non-significant (*p* > 0.05) alterations in the kidney functions as compared with the C group. However, CCl_4_ administration significantly (*p* < 0.05) increased urea and creatinine levels as compared to C group. In contrast, the administration of SE pre, during and/or after CCl_4_ administration significantly (*p* < 0.05) declined the urea and creatinine levels compared to CCl_4_ group.

##### Results summary

**Fig. 7:**
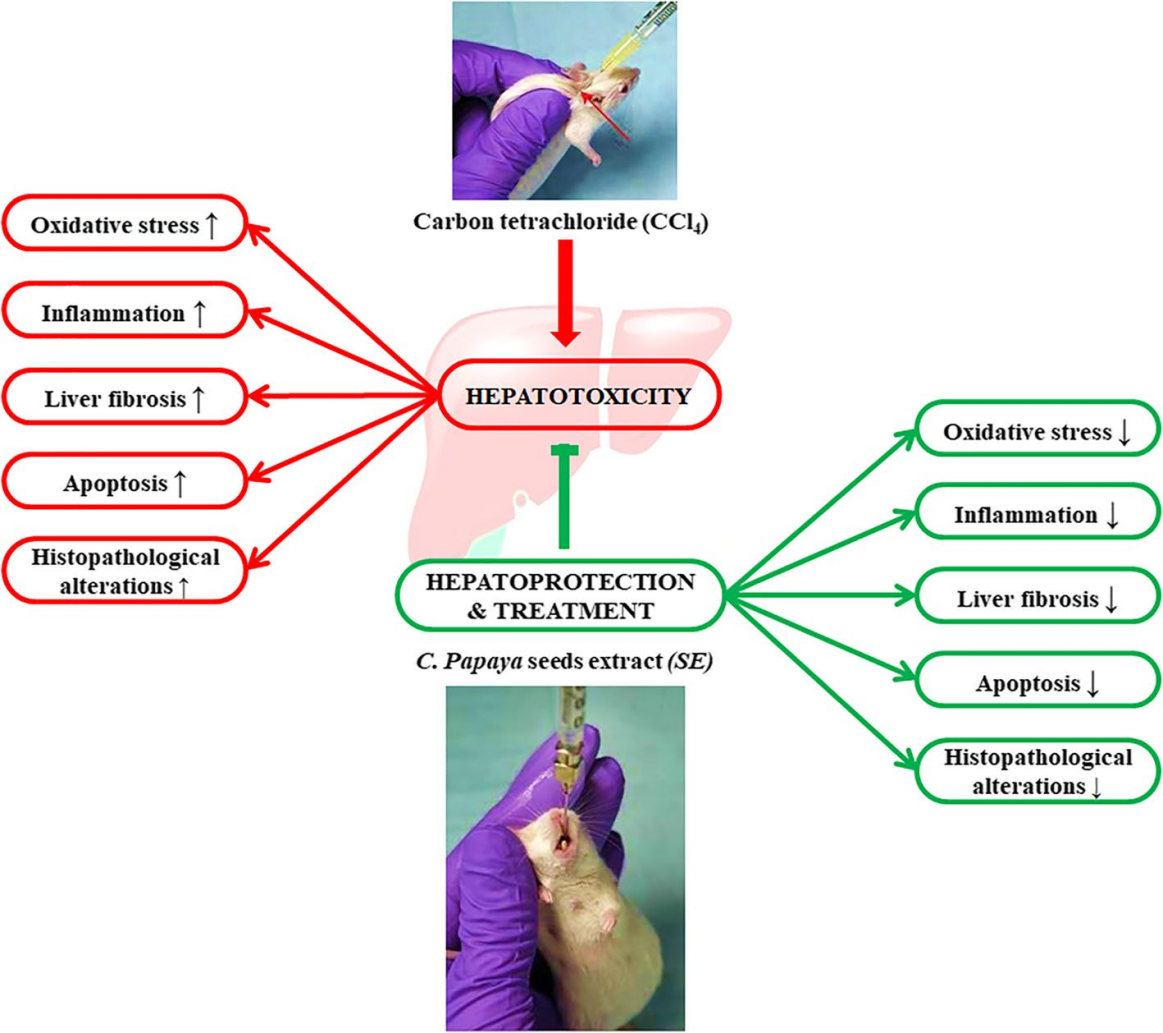
Graphical abstract

All results obtained in this study were summarized in Fig. 7.

## Discussion

The histological results of the present study revealed that CCl_4_ induced severe histological alterations in the hepatic tissue; extensive hepatocellular degenerations, fatty changes, and the presence of inflammatory cells. These results are in agreement with the biochemical results where CCl_4_ administration caused a significant elevation in MDA and NO levels beside GSR activity, with significant declines in GSH level and the activities of SOD, t-GPx and GST as compared to the control group. This indicates that CCl_4_ induced OS where it increased LP and disrupted the antioxidant system in the liver [56, 57]. In the liver, CCl_4_ is bio-transformed into active metabolites (CCl_3_• and CCl_3_OO•) which are responsible for the increment of the MDA level through the enhancement of LP of the polyunsaturated fatty acids of the cell membrane [58–60]. The elevation of NO level, a highly reactive molecule, may be related to the toxicity of CCl_4_ and its metabolites via stimulation of the inducible nitric oxide synthase which is responsible for NO synthesis [61–63]. The overproduction of NO inhibits the growth of lymphocytes and damages the surrounding cells, causing amplified inflammatory responses [62, 64]. Additionally, GSR maintains the redox condition by catalyzing the reduction of GSSG into GSH. So, the elevation of GSR activity after CCl_4_ administration represents a mechanism of adaptation to the induction of OS [10, 59]. GSH is responsible for the maintenance of redox homeostasis inside the cell. The reduction in GSH levels after CCl_4_ administration may be due to its consumption by t-GPX and GST [10]. Also, GSH depletion may be owed to the reaction between its SH-group and Cl_3_COO• (CCl_4_ metabolite) [65–67]. SOD stimulates the dismutation of superoxide radical (O_2_•¯) to form hydrogen peroxide (H_2_O_2_) [42]. Additionally, GPX, a selenoprotein enzyme, reduces H_2_O_2_ to H_2_O using GSH [68]. GST, an enzyme responsible for xenobiotics metabolism, catalyzes the conjugation of CCl_4_ and/or its metabolites with the SH-group of GSH, producing more water-soluble products [41, 63, 67]. In this study, the decreased in the SOD activity may be due to its inhibition through the interaction of CCl_4_ and its metabolites with the enzyme active site. Furthermore, the inhibition of the SOD activity may be due to the interference of the free radicals with the enzyme gene expression [10, 59]. The inhibition of t-GPX and GST may be related to the direct interaction of the free radicals on the enzyme molecules. Also, the depletion in the GSH level lead to the reduction of t-GPX and GST activities (Fig. 5) [10, 59]. The induction of LP of the mitochondrial membrane lipids after CCl_4_ administration causing the damage of cell integrity and increases the permeability of the membrane leading to cell death. Therefore, the protein biosynthesis was reduced and the hepatic enzymes were leaked into the blood circulation. Consequently, the levels of ALT, AST, and ALP in serum were increased while TP and albumin levels were decreased in rats injected with CCl_4_ as compared with the control group. Also, the liver damage changed lipid profile, where the LDL-c and TG levels were elevated while the HDL-c level was declined. Our results agree with the previous studies which reported that CCl_4_ induced hepatotoxicity [69].

On the other hand, the hepatic TNF-α, IL-6, NF-κB, TGF-β, and p53 gene expressions were up-regulated in rats after CCl_4_ administration. TNF-α and IL-6 are, pro-inflammatory cytokines, secreted from Kupffer cells (KCs) as response to liver damage induced by several xenobiotics such as CCl_4_ [10, 70]. The NF-κB, a transcription factor, plays a central role in inflammatory, immune, survival, and apoptosis progressions [71]. NF-κB is activated by a variety of inducers, including intercellular inflammatory cytokines, pathogen-derived substances, and many enzymes [72]. Stimulation of the inflammatory response plays a critical role in the pathology of CCl_4_-induced liver injury. The elevation in the TNF-α level stimulates the production of IL-6 and NF-κB which activates TGF-β synthesis, so the levels of NF-κB and TGF-β were increased as shown in Fig. 6. The elevation in TNF-α, IL-6, NF-κB and TGF-β lead to liver fibrosis. These results agreed with the previous studies which showed that the activated NF-κB stimulates both TGF-β signaling and hepatic stellate cells (HSCs) where the activated HSCs are transformed into myofibroblasts which in turn, increase collagen deposition in the extracellular matrix [73, 74].

The p53 is a tumor suppressor protein and a key sensor of the stress which induced in the cell such as OS, hypoxia, DNA damage, mitogenic oncogenes, and telomere shortening. The elevation in the level of p53 after CCl_4_ administration “as shown in Fig. 6” indicating that CCl_4_ induces hepatocytes apoptosis. Where, p53 triggers the apoptosis by increasing the pro-apoptotic gene expression and decreasing the anti-apoptotic genes [57, 75]. Previous studies reported that the promoter of the p53 gene has different binding sites one of them for p53 itself and another one for NF-κB since, the activation of NF-κB lead to up-regulation of p53 gene expression [60]. Moreover, the elevation of OS induced by CCl_4_ stimulates apoptosis via the elevation of gene expression of p53 and Bax and decreasing the gene expression of Bcl-2 and Bcl-xL [57, 75].

As well, CCl_4_ intoxication induced nephrotoxicity as the levels of creatinine and urea became greater than the normal values and these results are in accordance with the previous studies [10, 59].

Otherwise, the rats treated with SE pre, during, and/or after CCl_4_ administration showed significant improvements in the histological results of the liver. These results are in harmony with the biochemical results which revealed that SE treatment caused a significant reduction in the OS and LP induced by CCl_4_ and its reactive metabolites where MDA and NO levels were decreased consequently GSR activity was reduced, while the antioxidants, GSH, SOD, t-GPx, and GST were increased (Fig. 5). These results indicate that SE has antioxidant activity. Moreover, the results showed that SE had anti-inflammatory and anti-apoptotic powers, since the levels of TNF-α, IL-6, NF-κB, TGF-β, and p53 gene expressions, and NO level became lower than their corresponding values of the CCl_4_ group. The reduction of OS induced by CCl_4_ in rats treated with SE led to diminish of LP, inflammation, fibrosis and apoptotsis induced by CCl_4_ resulting in the reduction of liver damage and improving the liver functions and lipid profile. So the levels of AST, ALT, ALP, albumin, TP, HDL-c, LDL-c, and TG were improved as compared with CCl_4_ group. Otherwise, SE treatment reduced nephrotoxicity induced by CCl_4_ as shown from the improvement of kidney functions where the levels of creatinine and urea lower than those of CCl_4_.

The antioxidant, anti-inflammatory, and anti-apoptotic capacities of SE may be due to the beneficial effects of its contents which include phenolics, flavonoids, triterpenoids, tannins, and vitamin C which are present in large amounts as shown in Table 2. Additionally, the HPLC analysis of phenolic compounds in SE demonstrated the presence of different concentrations of catechol, gallic acid, caffeic acid, p-coumaric acid, o-coumaric acid, ferulic acid, and p-hydroxybenzoic acid. Also, the analysis of flavonoids revealed the occurrence of vanillin, RU, and quercitin with different concentrations (Fig. 2). Moreover, the previous studies showed that SE contains phytosterols and tocopherols [76]. All these compounds have antioxidant, anti-inflammatory, and anti-apoptotic potentials. Therefore, our results showed that SE contains considerable quantities of phenolic and flavonoid compounds. The antioxidant of SE may be related to the scavenging activities of phenolic and flavonoid compounds against ROS and RNS since these compounds are rich in hydroxyl groups [77, 78]. Therefore, SE has scavenging power against ABTS+, DPPH and FRAP (Fig. 3). These results were following the previous studies that confirm the antioxidant activities of SE [79]. Where, the phenolic and flavonoid compounds act as hydrogen or electron donors to stabilize the free radicals’ unpaired electrons, terminate the Fenton reaction, and prevent oxidative damage (Fig. 2 and Table 2) [59]. These results are in accordance with previous studies [79]. Tannins and triterpenoids in SE have also antioxidant capacity through chelating metal ions such as Fe (II) and interfering with the Fenton reaction; thereby they halt the OS [80, 81]. Additionally the presence of vitamin C in SE may amplify the antioxidant effect of the phenolic compounds. Phenolic and flavonoid compounds had anti-inflammatory effects CCl_4_-induced hepatotoxicity by decreasing the activation of NF-кB through the reduction of IкBα phosphorylation. The promoter of iNOS and TNF-α genes contain indeed binding sites for NF-кB, which could explain that SE extracts act on NO and TNF-α secretion, through an effect on NF-кB deactivations [82].

On the other hand, the data of the present study determined the presence of considerable amounts of important elements in SE. The existence of these elements, especially; S, Cu, Zn, Mn, and Se is considered as another path for the stimulation of the antioxidant system. S acts as an antioxidant element, which incorporated in GSH and thioredoxin synthesis [83]. Cu, Zn, and Mn are very important elements for the activity of SOD. Where SOD is presented in three isoforms; copper/zinc (Cu/Zn)-SOD, manganese (Mn)-SOD, and extracellular (EC)-SOD [47, 84, 85]. In addition, Zn itself act as antioxidant [22]. However, Se is a very important element in the protein synthesis of t-GPX and its activity [68].

Our results revealed that the administration of healthy rats with SE alone (400 mg/kg/day) for 12 weeks had no adverse effects, where there are no changes in OS, inflammation, liver and kidney functions when compared with the control group. Furthermore, SE administration reduced LDL-c level, and the markers of fibrosis (TGF-β) and apoptosis (p53) as compared with the control. The reduction of LDL-c may be owed to the effect of RU and kaempferol (componants of SE) which prevent the accumulation of lipids in the liver and decrease the level of LDL-c in the blood [86, 87]. Otherwise, the anti-fibrotic and anti-apoptotic activity of SE may be due to the presence of GA and caffeine, which play an important role in the down-regulation of collagen production in the hepatic stellate cells via the suppression of the expression of TGF-β and vascular endothelial growth factor (VEGF) [88, 89]. Additionally, quercetin down-regulates p53 expression, while Kaempferol inhibits the acetylation process of a target genes including poly [ADP-ribose] polymerase (PARP1), forkhead box protein (FOXO)-1, and p53 [90, 91].

## Conclusion

The present study revealed that the aqueous extract of *C. Papaya Linn.* seeds (SE) exhibited significant protective effects against CCl_4_-induced hepatotoxicity via reduction of OS, inflammation and apoptosis in hepatocytes induced by CCl_4_. This extract is promising agent for the inhibition of chemical - and drug-induced liver toxicity through improving the antioxidants and drug-metabolizing enzymes, decreasing the propagation of LP and improving of apoptosis.

## Data Availability

All data generated or analyzed during this study are included in this published article.

## Abbreviations

SE: *Carica Papaya Linn.* seeds extract
OS: Oxidative stress
CCl_4_: Carbon tetrachloride
GA: Gallic acid
UA: Ursolic acid
RU: Rutin
DNPH: 2,4 dinitrophenyl hydrazine
DPPH: 2,2 diphenyl-1-picrylhydrazyl
BHT: Butylated hydroxyltoluene
ABTS: 2,2-azinobis(3-ethylbenzothiazoline-6-sulfonic acid)
TBA: Thiobarbituric acid
MDA: Malondialdehyde
GSR: Glutathione reductase
GSH: Glutathione
SOD: Superoxide dismutase
GP_X_: Glutathione peroxidase
GST: Glutathione-S-transferase
TP: Total protein
ALT: Alanine aminotransferase
AST: Aspartate aminotransferase
ALP: Alkaline Phosphatase
LDL-c: Low-density lipoprotein cholesterol
HDL-c: High-density lipoprotein cholesterol
TG: Triglycerides
qRT-PCR: Quantitative real-time polymerase chain reaction
NF-κB: Nuclear factor kappa B
TNF-α: Tumor necrosis factor-α
IL-6: Interleukin-6
TGF-β: Transforming growth factor-β
